# Genistein supplementation modulates the intestinal microbiota–liver–lumbar muscle metabolic axis in DLY commercial pigs without concomitant changes in growth performance

**DOI:** 10.3389/fmicb.2025.1686257

**Published:** 2025-11-20

**Authors:** Yong Du, Yiting Yang, Yan Wang, Yuanyuan Wu, Chengming Liu, Ziling Hao, Zhijuan Yan, Junxiang Mao, Jiaqi Wen, Ye Zhao, Lili Niu, Xiaofeng Zhou, Linyuan Shen, Li Zhu, Mailin Gan

**Affiliations:** 1 Farm Animal Germplasm Resources and Biotech Breeding Key Laboratory of Sichuan Province, Sichuan Agricultural University, Chengdu, China; 2 State Key Laboratory of Swine and Poultry Breeding Industry, Sichuan Agricultural University, Chengdu, China; 3 Key Laboratory of Livestock and Poultry Multi-omics, Ministry of Agriculture and Rural Affairs, College of Animal and Technology, Sichuan Agricultural University, Chengdu, China; 4 Department of Biological Engineering, Sichuan Fisheries School, Chengdu, China

**Keywords:** genistein, 16SrRNA sequencing, liver metabolome, lumbar muscle metabolism, swine production

## Abstract

Genistein is widely found in leguminous plants. As an isoflavone, it has functions similar to mammalian estrogen and has effects on many aspects such as animal carcass performance, intestinal flora composition and physiological metabolism. This study aimed to investigate the effects of genistein supplementation on the carcass performance of commercial pigs via intestinal microorganisms, liver and lumbar muscle metabolites. Sixteen DLY commercial pigs were divided into two groups: a fed group receiving a diet supplemented with 160 mg/kg genistein and a control group without supplementation; the intervention lasted about 74 days. The bacterial genome of the 16SrRNA V3–V4 region was sequenced to observe the diversity and composition changes of microorganisms in the feces of the fed group and the control group. At the same time, the differential metabolites and metabolic pathways of the liver and lumbar muscle tissues of the fed group and the control group were jointly analyzed. The results of the study showed that the addition of genistein to feed can increase the relative abundance of *Firmicutes*, reduce the relative abundance of *Bacteroidetes*, and generally improve the relative abundance and diversity of intestinal flora. Combined analysis of intestinal microorganisms and liver and lumbar muscle metabolomes revealed that genistein supplementation can enhance the Aminoacyl-tRNA biosynthesis, Pentose phosphate pathway, and Pyrimidine metabolism through the gut-liver axis, promote the synthesis of proteins and nucleic acids, and maintain whole-body carbon–nitrogen metabolic homeostasis. It can also enhance Fructose and mannose metabolism, Amino sugar and nucleotide sugar metabolism, and Pentose phosphate pathway through the gut-muscle axis, promote the metabolism of carbohydrates such as amino sugars and fructose, provide energy, and maintain energy balance in the body. Correlation analysis showed that although there were no significant differences in host phenotypic values, genistein supplementation may affect host phenotypic values by changing the relative abundance of bacterial genera associated with the phenotype.

## 1 Introduction

Within the development of global animal husbandry, pork accounts for 35% of global meat consumption and is an important part of the dietary structure of many people (Gozalo-Marcilla et al., 2021). The number of countries involved in pig farming in the world is gradually increasing, and the total number of live pigs has shown explosive growth, which is mainly due to the improvement of breeding level and the advancement of breeding technology. On this basis, pork has surpassed beef and mutton to become the main livestock meat product consumed in the market (Chatellier, 2021). However, with the continuous improvement of living standards, people’s focus on pig farming has gradually shifted from the number of pigs to the quality of pigs. How to improve the production performance of pigs to achieve the goal of sustainable development has become one of the issues that current farming workers are thinking about. For example, computer machine learning algorithms can be used to accurately predict the farrowing performance of lactating sows, thereby assisting farmers in making feeding decisions and improving the farrowing performance of sows (Gauthier et al., 2022); Collect information on pig feeding behavior, feed intake, and weight, and use random forest and LASSO regression algorithms to predict the weight of pigs aged 159–166 days, thereby helping pork producers make wise marketing decisions for pigs while reducing labor and feeding costs (He et al., 2021). There are many ways to improve the production performance of pigs, from the nutritional level to the breeding level. Among the many methods to improve the production performance of breeding pigs, adding natural compounds that are beneficial to the growth and reproduction of pigs to feed is a cost-effective option.

Genistein is widely found in leguminous plants. As an isoflavone, it has functions similar to mammalian estrogen (Farmer et al., 2016). For example, the addition of genistein to the diet of growing sows resulted in proliferation of mammary parenchymal tissue after puberty, but there was no concomitant change in the expression of specific genes or circulating hormone levels in the mammary gland (Farmer et al., 2010). The estrogen level in animals not only affects the development and function of reproductive organs such as the ovaries, uterus, and breasts, but also affects the organs and tissues involved in the hypothalamus-pituitary-gonadal axis by binding to estrogen receptors. Injection of 440 mg/day of genistein in late gestation sows increased IGF1 concentration in sows and carcass fat content in newborn piglets (Farmer et al., 2016). The effects of soy isoflavones on the growth performance, intestinal morphology and antioxidant characteristics of weaned piglets were investigated. The results showed that long-term exposure to soy isoflavones can improve the growth performance of weaned piglets, protect intestinal morphology, and enhance antioxidant capacity (Li Y. P. et al., 2020). In the poultry field, the effect of adding genistein to the diet of chicks on the intestinal transcriptome was observed. The results showed that there were 3,281 upregulated genes and 3,851 downregulated genes in the feeding group. At the same time, the addition of genistein to the feed can improve the intestinal morphology, mucosal immune function, antioxidant activity and growth performance of chicks, thereby improving the growth performance of chicks with intestinal damage (Lv et al., 2020). Genistein was supplemented to laying-hen diets (50 mg/kg) to test its effects on systemic metabolism and egg quality. Treated hens exhibited improved glucose tolerance and yolk color, along with a significant enrichment of *Blautia, Ruminiclostridium* and *Clostridium sensu stricto 1* in the caecal microbiome, whose relative abundances correlated positively with plasma insulin sensitivity and eggshell strength (Obianwuna et al., 2024). These findings indicate that dietary genistein can modulate livestock gut microbiota and host metabolic indices beyond the murine model. In summary, genistein can play a role in many aspects in the livestock production process. It can promote animal meat and milk production, growth and development, and enhance cell proliferation and antioxidant capacity. It can also improve the balance of intestinal flora and maintain host health.

This study was designed to evaluate the impact of dietary genistein supplementation on 165-day-old Duroc × Landrace × Yorkshire (DLY) commercial fattening pigs. Using 16S rRNA gene sequencing (V3–V4 region), we first characterized changes in the relative abundance and composition of the intestinal microbiota. Subsequently, targeted metabolomics was applied to map metabolic alterations in both the liver and longissimus dorsi muscle. By integrating these multi-omics datasets, we aimed to elucidate how genistein-driven shifts in gut microbial communities influence host phenotypes through the gut–liver axis and gut–muscle axis, thereby providing a scientific basis for optimizing pig production performance.

## 2 Materials and methods

### 2.1 Ethics statement

All experimental procedures described below were approved by the Animal Ethical and Welfare Committee of Sichuan Agricultural University, Chengdu, China (Approval No. 2023302117 approval date: 1 July 2022).

### 2.2 Experimental animal slaughter and sample collection

The animals used in this study were provided by a farm in Sichuan Province, China. All animals had free access to food and water and were housed in similar conditions with an ambient temperature range of 28 to 37 °C. The diets met or exceeded the recommendations of the National Research Council (Lange, 2013) for crude protein, micronutrients, vitamins, and energy at different production stages. A total of 16 DLY commercial pigs were selected and randomly divided into two groups (8 pigs in each group): a fed group (GG) with genistein added to the feed and a control group (NC) without genistein. Among them, the added concentration of genistein in the fed group was 160 mg/kg. The complete ingredient composition and nutrient levels are now provided in Supplementary Table S1. After feeding the basal diet for 74 days, liver tissue and lumbar muscle tissue were collected from all pigs within 20 min after slaughter. The daily ration intake is shown in Supplementary Table S2. We use euthanasia methods on the animals during the slaughtering process and All pigs were transferred to a commercial slaughterhouse where the animals were stunned using a two-point electric shock technique, followed by bloodletting by trained staff following standard procedures that included inserting a knife into the ventral side of the base of the neck, in front of the sternum, and pointing it at the entrance to the rib cavity to cut off the blood vessels in the head and arm shafts, ensuring rapid blood loss of the animals (Pastrana-Camacho et al., 2025). Six commercial pigs with better health were selected from the two groups, and rectal feces were collected within 10 min after slaughter. All samples were immediately stored in liquid nitrogen tanks and returned to the laboratory and frozen in a −80 °C freezer for subsequent microbial and metabolomics analyses.

### 2.3 Phenotype data collection and 16S rRNA sequencing

Regarding growth performance, six commercial pigs in good health were weighed before slaughter. Backfat traits (in millimeters) were calculated by averaging measurements taken at three areas on the right side of the carcass (first rib, last rib, and last lumbar vertebra). The Body straight traits (in centimeters) are the straight length from the front edge of the pubic symphysis of the carcass to the shallow anterior part of the first cervical vertebra, and the Body oblique traits (in centimeters) are the straight length from the front edge of the pubic end to the tip of the first thoracic vertebra. The collected rectal stool samples were sent to Shanghai TianHao Biotechnology Co., Ltd. for total DNA extraction, DNA quality control, and library preparation. Each stool sample was sequenced in the V3–V4 region of the 16S rRNA gene using the Illumina NovaSeq platform (PE250).

### 2.4 Targeted metabolome analysis

The liver and lumbar muscle tissue samples were analyzed by liquid chromatography-mass spectrometry (LC–MS) technology of SHANGHAI BIOPROFILE TECHNOLOGY Co., Ltd. Specifically, Shimadzu Nexera X2 LC-30 AD system, equipped with ACQUITY UPLC HSS T3 column (1.8 μm, 2.1 × 50 mm column, Waters) and triple quadrupole mass spectrometer (5,500 QTRAP, AB SCIEX), metabolites were detected in electrospray negative ionization and positive ionization mode, and the mass spectrometry conditions were set as follows: negative ionization: source temperature 550 °C, ion source gas 1 (GAS1): 40, ion source gas 2 (GAS2): 50, curtain gas (CUR): 35, ion spray voltage fluctuation (ISVF): −4,500 V; positive ionization: source temperature 550 °C, ion source gas 1 (GAS1): 40, ion source gas 2 (GAS2): 50, curtain gas (CUR): 35, ion spray voltage floating (ISVF): 5500 V. The MRM (multiple reaction monitoring) mode was adopted to detect the transitions. The raw MRM data of MT1000 KIT metabolites were extracted using MultiQuant 3.0.2 software, and the peak area of each metabolite was subsequently obtained. The discriminant metabolites were obtained using the statistically significant threshold of the variable projection (VIP) values obtained from the OPLS-DA model and the two-tailed Student’s *t*-test (*p*-value) of the normalized raw data. Metabolites with VIP greater than 1 and *p*-value less than 0.05 were considered statistically significant metabolites.

### 2.5 Statistical analysis

The 16SrRNA data were analyzed using QIIME2 (v2024.5) software and its integrated plug-ins. First, the single-end 16S rRNA sequencing data was imported using the QIIME tools import plug-in. The raw data was then denoised and filtered using the QIIME dada2 denoise-paired plug-in and the QIIME feature-table filter-features plug-in. The QIIME phylogeny align-to-tree-mafft-fasttree plug-in was then used to construct a phylogenetic tree of the filtered data. Alpha diversity and beta diversity analysis were performed using the QIIME Diversity Core-Metrics plug-in. Species annotation was performed using the QIIME Feature-Classifier plug-in. Finally, PICRUSt2 (v2.5.3) was used to predict the function of the OTU abundance table. The microeco package in R language was used to draw dilution curves, perform species composition analysis, and count species diversity (Liu et al., 2021). The Lefse package in R language was used to annotate differential microbiota, set the LDA value to be greater than or equal to 2 to screen differential bacterial genera, and calculate the Spearman correlation between intestinal flora and economic traits at the genus level (Khleborodova et al., 2024). The *T*-test difference analysis method in stamp software was used to find differential metabolic pathways (Parks et al., 2014). SPSS software (26.0) was used for phenotypic difference analysis, and the difference was considered significant when *p* < 0.05.

## 3 Results

### 3.1 Effects of genistein supplementation on phenotype traits of DLY commercial pigs

**Figure 1:**
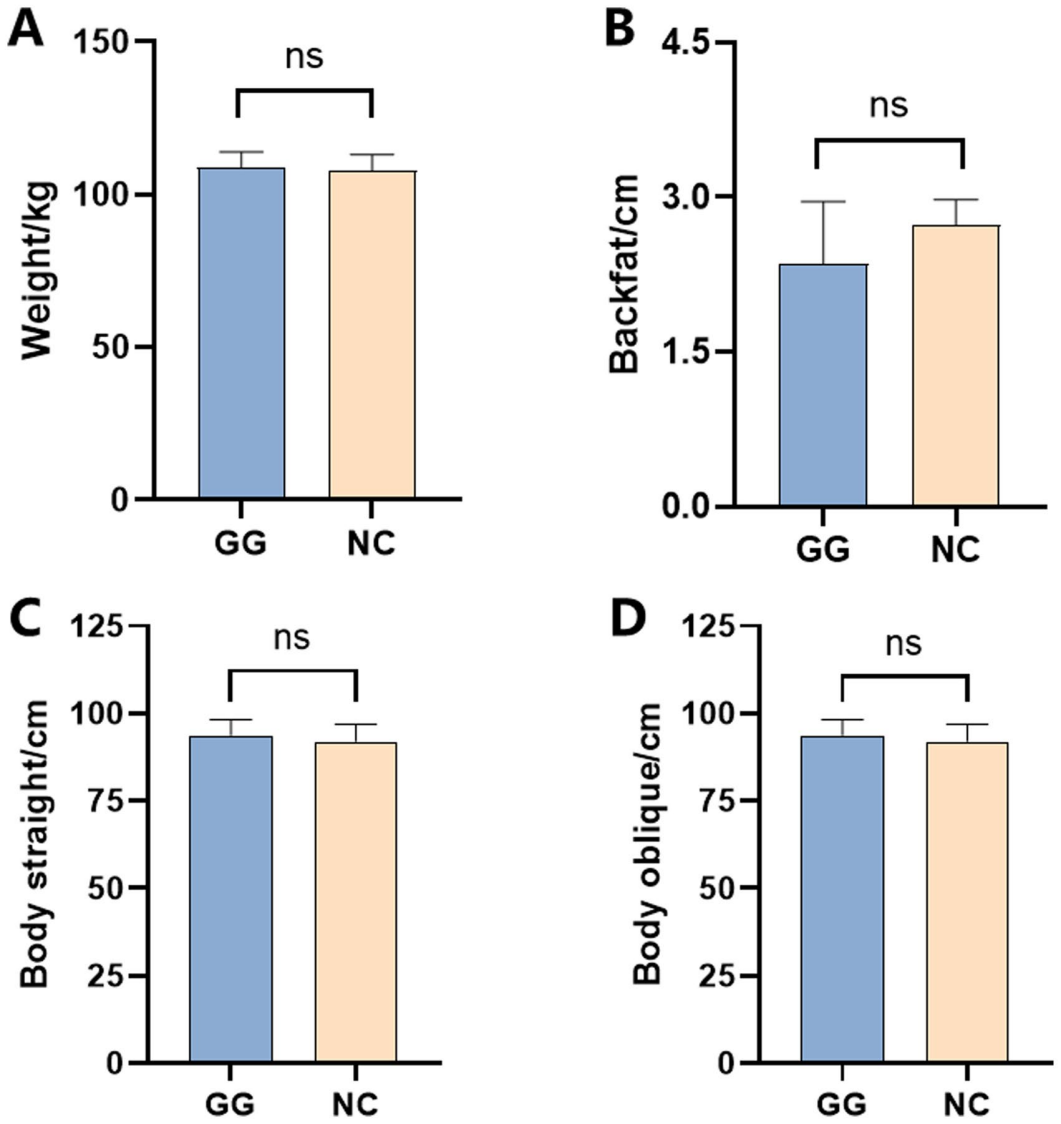
Comparison of the phenotypic values of weight, backfat, body straight, and body oblique between the fed group and the control group. **(A–D)** The phenotypes of weight, backfat, body straight, and body oblique, respectively.

Based on the phenotypic determination and *T*-test results of the fed group and the control group, as shown in Table 1, compared with the control group, the Weight of the fed group decreased by approximately 11.66 kg (Figure 1A), the Backfat decreased by approximately 0.35 mm (Figure 1B), the Body straight increased by approximately 1 cm (Figure 1C), and the Body oblique length increased by approximately 1.83 cm (Figure 1D). There were no significant differences in phenotypic traits between the fed group and the control group under *T*-test (*p* > 0.05).

**Table 1:** Comparison of the effects of genistein supplementation on phenotypic traits of DLY commercial pigs.

Group	GG	NC	*P* value
Weight/kg	**133.67 ± 14.32**	**145.33 ± 11.35**	**0.15**
Body straight/cm	**108.83 ± 5.15**	**107.83 ± 5.23**	**0.75**
Body oblique/cm	**93.83 ± 4.36**	**92.00 ± 4.90**	**0.51**
Backfat/mm	**2.35 ± 0.60**	**2.73 ± 0.25**	**0.19**

Bold values indicate the mean ± standard deviation. Values in bold differ significantly between groups at *p* < 0.05.

### 3.2 Effects of genistein supplementation on intestinal flora diversity in DLY commercial pigs

**Figure 2:**
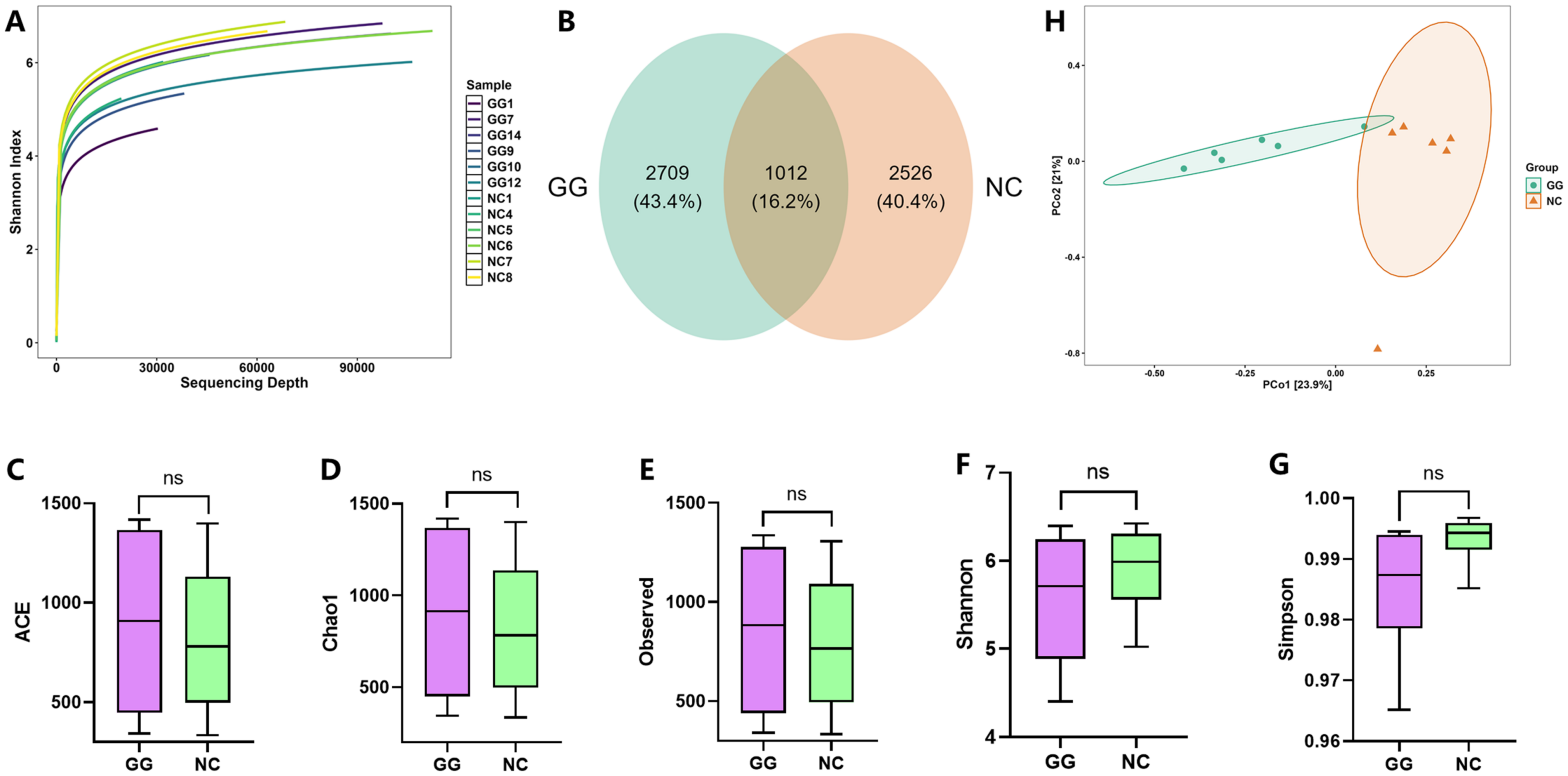
Analysis of intestinal microbial diversity between the fed group and the control group. **(A)** Rarefaction curve of samples in the fed group and the control group; **(B)** Venn diagram of OTU number between the fed group and the control group; **(C–G)** Comparison of alpha diversity index between the fed group and the control group, from left to right are ACE index, Chao1 index, Observed index, Shannon index, Simpson index; **(H)** PCoA distribution diagram of beta diversity.

Based on the 16S microbiome sequencing data, the Rarefaction curve of the fed group and the control group samples showed similar trends. Most samples first rose rapidly and then flattened, but the sequencing depth of the three samples GG1, NC1 and NC4 was low (Figure 2A). Overall, the width of the curve in the fed group was not much different from that in the control group. After quality control and grouping, a total of 6,247 OTU units were observed (Figure 2B), of which 2,709 independent OTU units were detected in the fed group samples, accounting for 43.4%, and 2,526 independent OTU units were detected in the control group samples, accounting for 40.4%. The number of OTU units shared by the two was 1,012, accounting for 16.2%.

The results of alpha diversity analysis showed that compared with the control group, the ACE index, Chao1 index, and Observed index of the fed group increased, but the Simpson index and Shannon index decreased. Wilcoxon analysis showed that there was no significant difference in ACE index, Chao1 index, Observed index, Simpson index, and Shannon index between the fed group and the control group (Figures 2C–G). The results of beta diversity analysis showed that the data of the control group and the fed group were relatively concentrated, and the distance between the two groups was generally far, with good dispersion (Figure 2H). PCo1 could explain 23.9% of the variation between the groups, PCo2 could explain 21% of the variation, and PERMANOVA analysis showed that there was a very significant difference in the microbial community structure between the two groups (*p* = 0.003).

### 3.3 Effects of genistein supplementation on intestinal microbial differential flora in DLY commercial pigs

**Figure 3:**
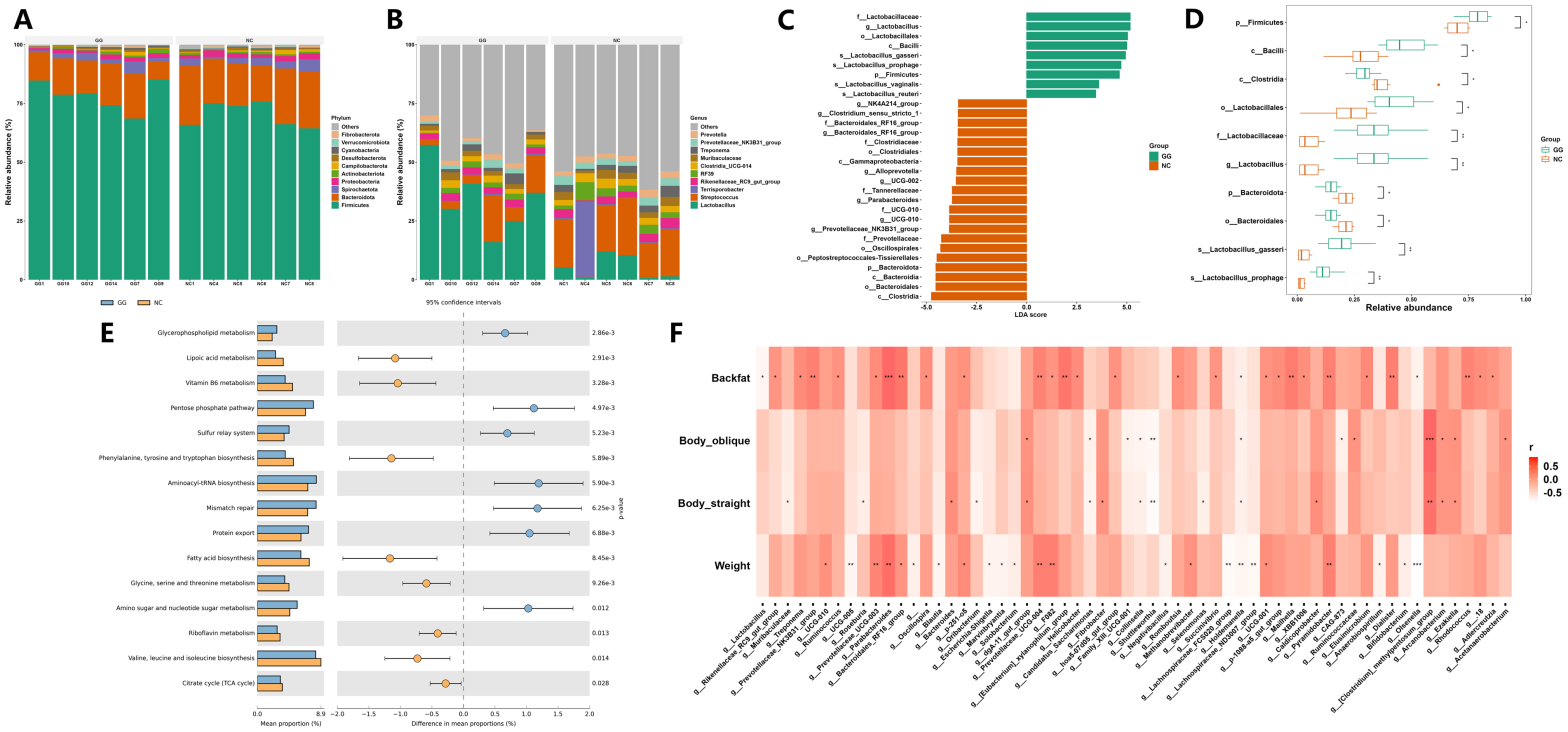
Differential bacterial genus distribution and genus phenotype correlation between the fed group and the control group. **(A)** The top ten intestinal flora in relative abundance at the phylum level between the fed group and the control group; **(B)** The top ten intestinal flora in relative abundance at the genus level between the fed group and the control group; **(C,D)** Comparison of different bacterial communities and their relative abundance between the fed group and the control group using Lefse analysis; **(E)** KEGG enriched differential functional pathways between the fed group and the control group; **(F)** Distribution of spearman correlations between gut microbiota and phenotypes at the genus level.

Based on the analysis of the intestinal microbial species richness of the DLY commercial pigs in the fed group and the control group at the phylum level, the results showed that the top three microorganisms in the two groups were *Firmicutes, Bacteroidota*, and *Spirochaetota*. The three together accounted for more than 90% at the phylum level and were the main intestinal microorganisms at the phylum level. Compared with the control group, the relative abundance of *Firmicutes* in the fed group increased by about 8.4%, the relative abundance of *Bacteroidetes* decreased by 6.5%, and the relative abundance of *Spirochetes* remained basically unchanged (Figure 3A). The intestinal microbial species richness of DLY commercial pigs in the fed group and the control group was analyzed at the genus level. The results showed that the main genera between the two groups were *Lactobacillus*, *Streptococcus*, and *Rikenbacteriaceae RC9 enteric* group. Compared with the control group, the relative abundance of *Lactobacillus* in the fed group increased by about 28.91%, the relative abundance of *Streptococcus* decreased by about 8.04%, and the RC9 intestinal flora of the *Rikenbacteriaceae family* remained basically unchanged (Figure 3B). The results of Lefse differential analysis showed that the main differences in intestinal microorganisms between the fed group and the control group were concentrated in the *Firmicutes* and *Bacteroidetes* (Figure 3C). Compared with the control group, the relative abundance of *Lactobacillus*, *Lactobacillus_gasseri*, and *Lactobacillus_prophage* from the *Lactobacillaceae family* in the *Firmicutes* increased in the fed group with extremely significant differences (*p* < 0.01).

The relative abundance of *UCG-010* from the *UCG-010 family, Clostridium_sensu_stricto_1* from the *Clostridiaceae family, Parabacteroides,* and *Prevotellaceae_NK3B31_group* in the *Bacteroidetes* decreased in the fed group with significant differences (*p* < 0.05) (Figure 3D). The results of KEGG database function prediction combined with *T*-test difference analysis showed that a total of 35 KEGG functional difference pathways were found, and there were significant differences between the fed group and the control group (*p* < 0.05). In the top pathway classification, it is related to Genetic Information Processing, Cellular Processes, and Metabolism, and most of the pathways are mainly enriched in Metabolism. In the metabolic process, it is mainly related to biological processes such as Glycan biosynthesis and metabolism, Carbohydrate metabolism, Amino acid metabolism, Lipid metabolism, Metabolism of cofactors and vitamins, Metabolism of other amino acids, Energy metabolism, Nucleotide metabolism, and Metabolism of terpenoids and polyketides in the second pathway. At the third pathway, it is mainly related to Histidine metabolism, Valine, leucine and isoleucine biosynthesis, Glycine, serine and threonine metabolism, Phenylalanine, tyrosine and tryptophan biosynthesis, Citrate cycle (TCA cycle), etc. In addition, we also noticed that processes such as Amino sugar and nucleotide sugar metabolism, Aminoacyl-tRNA biosynthesis, and Mismatch repair were mainly enriched in the fed group, while processes such as Fatty acid biosynthesis, Phenylalanine, tyrosine and tryptophan biosynthesis, and Vitamin B6 metabolism were mainly enriched in the control group (Figure 3E).

The results of Spearman correlation analysis between intestinal microorganisms and phenotypes showed that at the genus level, *Olsenella, Parabacteroides, Lachnospiraceae_FCS020_group, Pyramidobacter, Holdemanella* and other bacterial genera showed extremely significant correlation with Weight (*p* < 0.01). *Parabacteroides, Mailhella, Dialister, Rhodococcus* and other genera showed extremely significant correlation with Backfat traits (*p* < 0.01). *Shuttleworthia, Candidatus_Saccharimonas, Collinsella* and other genera showed significant correlation with Body straight and Body oblique (*p* < 0.05) (see Figure 3F).

### 3.4 Effects of genistein supplementation on liver metabolites in DLY commercial pigs

**Figure 4:**
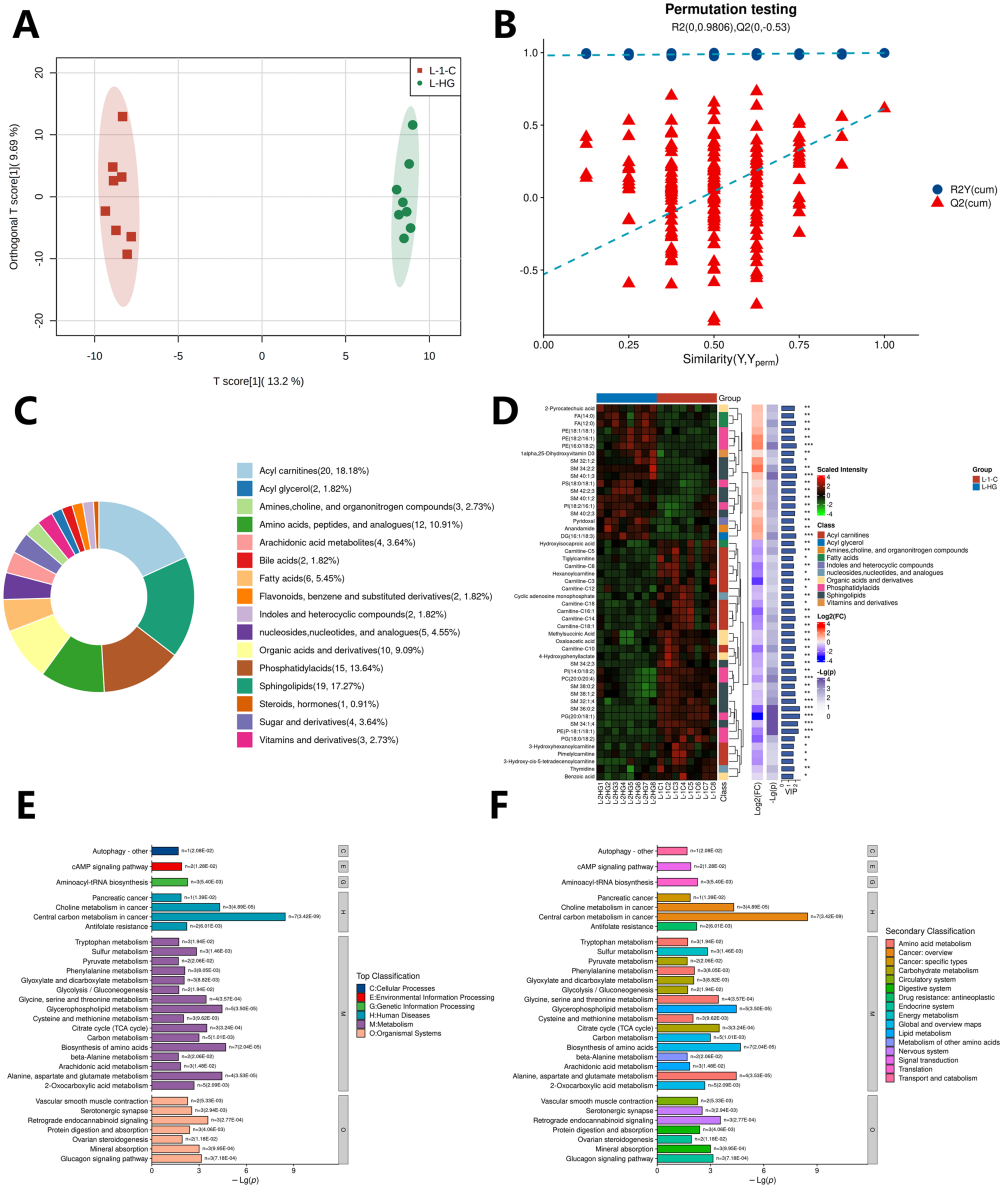
Metabolome analysis of DLY commercial pig livers in the fed group and the control group. **(A)** Cluster diagram of OPLS-DA model of samples between the fed group and the control group; **(B)** Permutation test of OPLS-DA model between the fed group and the control group; **(C)** Main classification of DLY commercial pig liver metabolites; **(D)** Heatmap of the top 50 significantly different metabolites in DLY commercial pig liver; **(E)** Distribution of KEGG enriched top pathways of DLY commercial pig liver metabolites; **(F)** Distribution of KEGG enriched secondary pathways of DLY commercial pig liver metabolites.

The results of the OPLS-DA model combined with the permutation test showed that the liver metabolites of the fed group and the control group had large differences between the groups in the OPLS-DA model, and the intra-group clustering effect was good (Figure 4A). The R2Y(cum) and Q2(cum) of the permutation test were 0.998 and 0.615, respectively. The intercepts of R2 and Q2 were 0.981 and −0.53 (Figure 4B), indicating that the model was not overfitted and was relatively reliable. There were significant differences in liver metabolites between the fed group and the control group.

The results of OPLS-DA model combined with volcano plot showed that a total of 110 significantly differential metabolites were identified between the fed group and the control group, including 72 down-regulated differential metabolites and 38 up-regulated differential metabolites, which can be divided into about 16 categories of metabolites, including Phosphatidylacids, Acyl carnitines, Sphingolipids, Organic acids and derivatives, Bile acids, Amino acids, peptides, and analogues, Vitamins and derivatives, Fatty acids, Indoles and heterocyclic compounds, Acyl glycerol, Sugar and derivatives, Flavonoids, benzene and substituted derivatives, nucleosides, nucleotides, and analogues, Amines, choline, and organonitrogen compounds, Steroids, hormones, Arachidonic acid metabolites, etc. (Figure 4C). The top 50 metabolites with significant differences in VIP values were selected to draw a heatmap. The results showed that compared with the control group, the concentrations of metabolites such as PE (16:0/18:2), SM 40:1;3, and DG (16:1/18:3) increased significantly (*p* < 0.01), while the concentrations of metabolites such as PC (20:0/20:4), SM 32:1;4, and PG (20:0/18:1) decreased significantly (*p* < 0.01) (see Figure 4D).

The enrichment results of KEGG database showed that 87 KEGG functional pathways were enriched in 110 significantly differential metabolites, and the top 30 functional pathways were selected for visualization based on the significance ranking. The results showed that the differential metabolites between the fed group and the control group were mainly related to Metabolism, Human Diseases, Organic Systems, Cellular Processes, Environmental Information Processing, and Genetic Information Processing in the top pathway (Figure 4E). In the secondary pathway, it is mainly related to Amino acid metabolism, Carbohydrate metabolism, Cancer: overview, Cancer: specific types, Circulatory system (Figure 4F).

### 3.5 Effects of genistein supplementation on metabolites of commercial pig lumbar muscle

**Figure 5:**
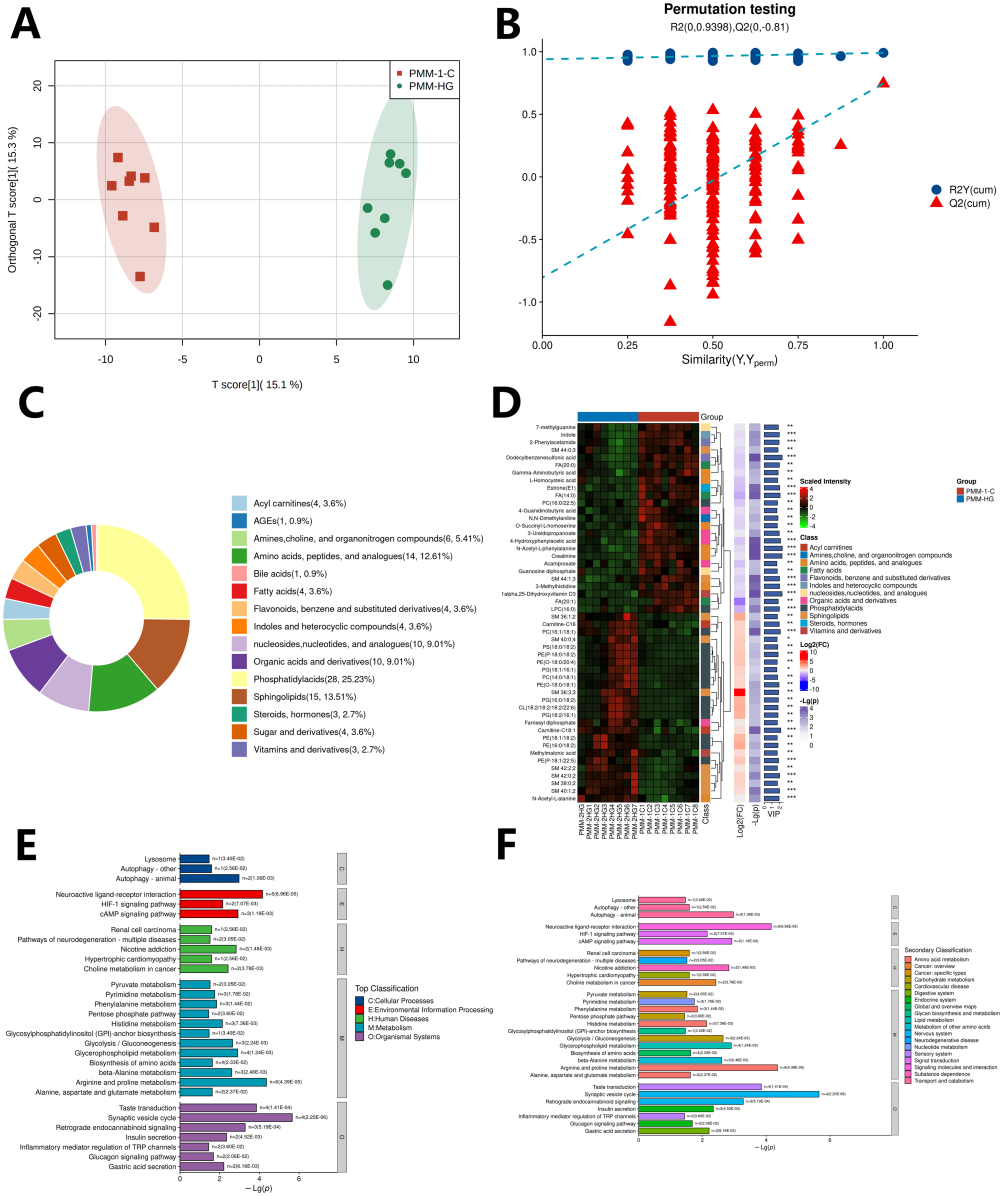
Metabolome analysis of DLY commercial pig lumbar muscle in the fed group and the control group. **(A)** Cluster diagram of the OPLS-DA model of samples between the fed group and the control group; **(B)** OPLS-DA model permutation test between the fed group and the control group; **(C)** Main classification of metabolites in commercial pig lumbar muscle; **(D)** Heatmap of the top 50 significantly different metabolites in commercial pig lumbar muscle; **(E)** KEGG enrichment of top pathways of metabolites in commercial pig lumbar muscle; **(F)** KEGG enrichment of secondary pathways of metabolites in commercial pig lumbar muscle.

The results of the OPLS-DA model combined with the permutation test showed that the metabolites of the lumbar muscle between the fed group and the control group had large differences between the groups in the OPLS-DA model, and the intra-group clustering effect was good (Figure 5A). The R2Y(cum) and Q2(cum) of the permutation test were 0.991 and 0.746, respectively. The intercepts of R2 and Q2 were 0.94 and −0.805 (Figure 5B), indicating that the model was not overfitted and was relatively reliable. There were significant differences in the metabolites of the lumbar muscle between the fed group and the control group.

The results of OPLS-DA model combined with volcano plot showed that a total of 111 significantly differential metabolites were identified between the fed group and the control group, including 58 down-regulated differential metabolites and 53 up-regulated differential metabolites, which can be divided into about 15 categories of metabolites, including fatty acids, phosphatidylacids, sugar and derivatives, stereoids, hormones, organic acids and derivatives, biles acids, amino acids, peptides, and analogues, flavonoids, benzene and substituted derivatives, AGEs, sphingolipids, vitamins and derivatives, indoles and heterocyclic compounds, nucleosides, nucleotides, and analogues, amines, choline, and organonitrogen compounds, and aceyl carnitines (Figure 5C). The top 50 metabolites with significant differences in VIP values were selected to draw a heatmap. The results showed that compared with the control group, the concentrations of metabolites such as indole, 2-Phenylacetamide, and Dodecylbenzenesulfonic acid decreased significantly (*p* < 0.01), while the concentrations of metabolites such as PC (16:1/18:1) and Carnitine-C18:1 increased significantly (*p* < 0.01) (see Figure 5D).

The enrichment results of KEGG database showed that 99 KEGG functional pathways were enriched in 111 significantly differential metabolites, and the top 30 functional pathways were selected for visualization based on the significance ranking. The results showed that the differential metabolites between the fed group and the control group were mainly related to Metabolism, Human Diseases, Organic Systems, Cellular Processes, and Environmental Information Processing in the top pathway (Figure 5E). In the secondary pathway, it is mainly related to Amino acid metabolism, Glycan biosynthesis and metabolism, Transport and catabolism, Nucleotide metabolism, Lipid metabolism and other pathways (see Figure 5F).

### 3.6 Combined analysis of intestinal flora and liver and lumbar muscle metabolism

**Figure 6:**
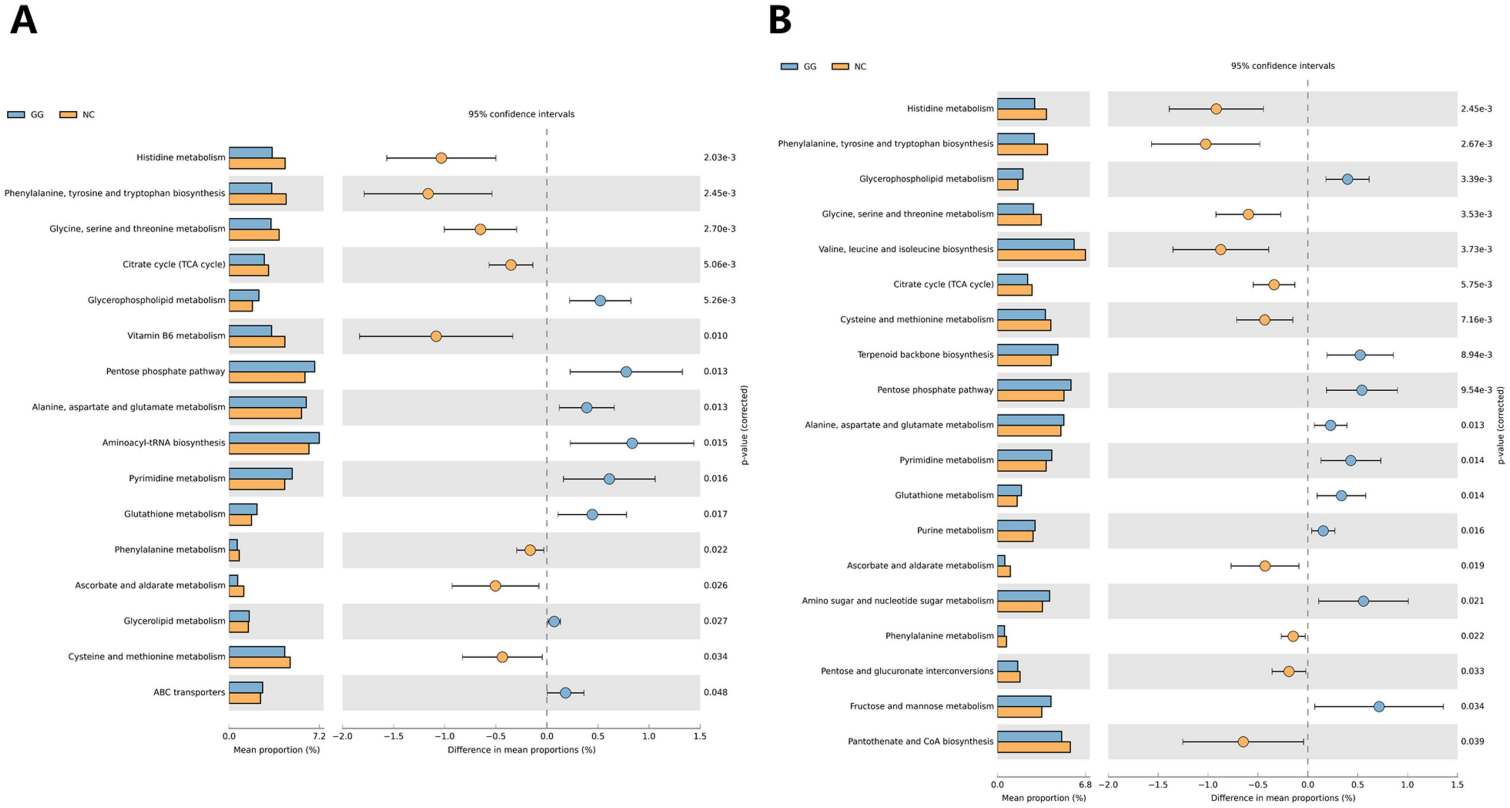
Combined analysis of functional pathways of intestinal flora and liver and lumbar muscle metabolome. **(A)** Comparison of differential pathways in joint analysis of intestinal flora and liver metabolome; **(B)** Comparison of differential pathways in joint analysis of intestinal flora and lumbar muscle metabolome.

The results of the joint analysis of the differential functional pathways of intestinal microorganisms and liver found that there were 42 common pathways between intestinal microorganisms and liver metabolomes, which were mainly related to Metabolism, Genetic Information Processing, Environmental Information Processing, Organic Systems, and Human Diseases in the primary pathways. In the secondary pathway, it is mainly related to Amino acid metabolism, Carbohydrate metabolism, Lipid metabolism and other pathways. *T*-test analysis results showed that 16 pathways shared by intestinal microorganisms and liver metabolomes showed significant differences (*p* < 0.05), and the top five extremely significant pathways (*p* < 0.01) were Histidine metabolism, Phenylalanine, tyrosine and tryptophan biosynthesis, Glycine, serine and threonine metabolism, Citrate cycle (TCA cycle), and Glycerophospholipid metabolism. It is worth noting that the enrichment of pathways such as Histidine metabolism, Phenylalanine, tyrosine and tryptophan biosynthesis, and Vitamin B6 metabolism in the fed group was significantly lower than that in the control group, while the enrichment of pathways such as Aminoacyl-tRNA biosynthesis, Pentose phosphate pathway, and Pyrimidine metabolism was significantly higher than that in the control group (see Figure 6A).

The results of the joint analysis of the differential functional pathways of intestinal microorganisms and lumbar muscles showed that there were 44 pathways in common between intestinal microorganisms and lumbar muscle metabolomes, which were mainly related to Metabolism, Environmental Information Processing, Cellular Processes, and Organic Systems in the primary pathways. In the secondary pathways, they were mainly related to metabolic pathways such as Carbohydrate metabolism, Amino acid metabolism, Lipid metabolism, and Metabolism of cofactors and vitamins. *T*-test analysis results showed that 19 pathways shared by intestinal microorganisms and lumbar muscle metabolome showed significant differences (*p* < 0.05), and the top nine extremely significant pathways (*p* < 0.01) were Histidine metabolism, Phenylalanine, tyrosine and tryptophan biosynthesis, Glycine, serine and threonine metabolism, Glycerophospholipid metabolism, Valine, leucine and isoleucine biosynthesis, Cysteine and methionine metabolism, Citrate cycle (TCA cycle), Terpenoid backbone biosynthesis, and Pentose phosphate pathway. It is worth noting that in the fed group, the enrichment levels of Histidine metabolism, Phenylalanine, tyrosine and tryptophan biosynthesis, Valine, leucine and isoleucine biosynthesis were significantly lower than those in the control group, while the enrichment levels of Fructose and mannose metabolism, Amino sugar and nucleotide sugar metabolism, and Pentose phosphate pathway were significantly higher than those in the control group (see Figure 6B).

## 4 Discussion

In the process of animal husbandry, carcass performance evaluation is crucial to improving pig production efficiency, and it can well reflect the production performance and economic benefits of pigs (Zhou et al., 2025). For example, Backfat traits can affect the reproductive performance of sows. Its importance lies in the high correlation with the nutritional status, lactation performance and overall health of the sows themselves (Mun et al., 2023). Genistein, as the aglycone form of soybean isoflavones, has higher antioxidant activity (Li Y. et al., 2024). It is beneficial to improve the growth performance of weaned piglets, protect intestinal morphology, and improve antioxidant capacity (Li Y. P. et al., 2020). Our research results show that although it is not statistically significant, the Body straight and Body oblique are slightly improved, and the body weight and backfat are slightly decreased after feeding with genistein, and there is a trend of affecting the traits. Next, we used 16S rRNA sequencing technology to explore the effect of genistein supplementation on the diversity of intestinal flora in fattening pigs. The rarefaction curve can directly reflect the rationality of the sequencing data and indirectly reflect the relative abundance of species in the sample (Huse et al., 2008). The alpha diversity index can directly reflect the richness and diversity of the microbial community in the sample (Peterson et al., 2021). Beta diversity can be used to compare the composition of microbial communities in different samples, reflecting whether there are community differences between different samples (Lkhagva et al., 2021). Our results show that the rarefaction curves of most samples first rise rapidly and then flatten, but the sequencing depth of the three samples GG1, NC1 and NC4 is low. Overall, the width of the curve of the fed group is not much different from that of the control group. Combining the comparison of the number of OTU classification units and alpha and beta diversity between the two, it was found that the addition of genistein to feeding can slightly increase the species relative abundance of intestinal flora and change the composition ratio of the microbial community, but it does not affect the uniformity of species distribution.

Consistent with most studies, *Firmicutes* and *Bacteroidetes* were the dominant phyla in both the fed and control groups (Kumar et al., 2020; Li X. J. et al., 2020). However, in the fed group, the relative abundance of *Firmicutes* increased significantly, the relative abundance of *Bacteroidetes* decreased significantly, and the relative abundance of other phyla remained basically unchanged. *Firmicutes* are Gram-positive bacteria, mainly from the genera *Bacillus* and *Clostridium*, which play a key role in the host’s nutrition and metabolism through the synthesis of short-chain fatty acids. *Firmicutes* bacteria can also indirectly connect to other tissues and organs through their metabolites to regulate hunger and satiety. They have many genes responsible for fermenting dietary fiber and can interact with the intestinal mucosa to promote homeostasis (Sun et al., 2023). *Bacteroidetes* are gram-negative bacteria, mainly from the genera *Bacteroides, Parabacteroides* and *Prevotella*. Their components lipopolysaccharide and flagellin interact with cell receptors and enhance immune responses through cytokine synthesis (Liu et al., 2024; Stojanov et al., 2020). The KEGG database is used to understand the higher-level functions and uses of cells, organisms, and ecosystems from information at the molecular level, especially from large-scale molecular data sets generated by genome sequencing and other high-throughput experimental technologies (Kanehisa et al., 2019). Amino sugar and nucleotide sugar metabolism pathways involve the synthesis and metabolism of amino sugars and nucleotide sugars, and their abnormalities are associated with a variety of diseases (Lee et al., 2016). Aminoacyl-tRNA biosynthesis is a key step in protein synthesis and plays a role in biosynthetic pathways such as cell wall synthesis, antibiotic synthesis, lipid modification and protein degradation (Ibba and Söll, 2001). Mismatch repair is a DNA repair mechanism that corrects base mismatches produced during DNA replication, maintains genome stability, and helps prevent mutations and cancer (Gao et al., 2021). The fatty acid biosynthesis pathway is a process in which fatty acids are produced from acetyl coenzyme and NADPH through the action of fatty acid synthase. Its regulation is crucial for maintaining the energy balance and cell function of organisms (Song et al., 2024). The phenylalanine, tyrosine and tryptophan biosynthesis pathway mainly involves the synthesis of three essential amino acids: phenylalanine, tyrosine and tryptophan, which are crucial for maintaining cell structure and function (Wiggins et al., 2015). Vitamin B6 metabolism pathway involves the synthesis and decomposition of vitamin B6. The active form of vitamin B6 participates in the biosynthesis and decomposition of amino acids and the biosynthesis of fatty acids through the action of a series of enzymes. It plays an important role in maintaining the integrity of cell membranes and protecting cells from oxidative damage (Mayengbam et al., 2020). Our results show that genistein supplementation can enhance the synthesis and metabolism of amino sugar and nucleotide sugar through amino sugar and nucleotide sugar metabolism and mismatch repair pathways, correct base mismatches produced during DNA replication, reduce the probability of gene mutation and cancer, and reduce fat deposition by weakening the fatty acid biosynthesis pathway.

Spearman correlation can determine the simple linear relationship between two variables under dimensionless measurement and can be used to calculate the correlation coefficient between different bacterial genera and host phenotypes (Tzeng et al., 2021). Through the calculation of Spearman correlation coefficient, we observed that at the genus level, *Olsenella* was significantly negatively correlated with Body weight (r = −0.85), *Parabacteroides* was significantly positively correlated with Body weight (r = 0.82), *Parabacteroides* was significantly positively correlated with backfat (r = 0.83), and *Lactobacillus* was significantly negatively correlated with backfat (r = −0.69). As a pair of highly correlated traits, Body straight and Body oblique are mostly the same genus of bacteria that show significant correlation with the two (Li R. et al., 2024). For example, the genus *Shuttleworthia* showed a highly significant negative correlation with the Body straight and the Body oblique (r = −0.79, r = 0.73), and the *genus [Clostridium]_methylpentosum_group* showed a highly significant positive correlation with the body straight and the body oblique (r = 0.83, r = 0.81). Although there were no significant differences in the host phenotypic values, the Body straight and Body oblique of the fed group showed an upward trend, while the Body weight and backfat showed a downward trend compared with the control group. This indicates that the addition of genistein to feeding may affect the host phenotypic values by changing the relative abundance of bacterial genera related to the phenotype. For example, the metabolic activity of *Parabacteroides* may enhance fat deposition, leading to increased backfat thickness (Chen et al., 2023). *Lactobacillus reuteri* from Ningxiang pigs can regulate host intramuscular fat deposition by regulating branched-chain amino acid metabolism, thereby reducing backfat thickness (Yang et al., 2025).

The OPLS-DA model is a supervised discriminant analysis statistical method that uses partial least squares regression to establish a relationship model between metabolite expression and sample category to predict sample category. It has a wide range of applications in metabolomics analysis (Thevenot et al., 2015). Our results showed that there was a clear separation between the fed group and the control group for both the liver metabolome and the lumbar muscle metabolome, and significant differences were found between the endogenous metabolites. The gut-liver axis refers to a bidirectional signaling network between the intestine and the liver, which plays an important role in regulating the body’s glucose and lipid metabolism, inflammatory response, and maintaining liver and intestinal function (Tilg et al., 2022). The gut-muscle axis refers to the interaction between the intestine and skeletal muscle, which influences muscle health and function through multiple mechanisms (Mancin et al., 2023). Since the intestinal flora affects the metabolic processes of the liver and muscles through the gut-liver axis and the gut-muscle axis, the functional pathways shared by the intestinal microorganisms and the liver and lumbar muscle metabolism can further explain how the intestinal flora affects the metabolic state of the liver and muscles through metabolite and signal analysis (Wang et al., 2025). Histidine metabolism mainly involves the synthesis and decomposition of histidine. One of its metabolites, histamine, plays an important role in immune response, inflammation and neurotransmission (Brosnan and Brosnan, 2020). Pentose phosphate pathway is an important pathway in cell metabolism, mainly responsible for the generation of ribose-5-phosphate and NADPH, which plays an important role in maintaining whole-body carbon–nitrogen metabolic homeostasis (TeSlaa et al., 2023). Pyrimidine metabolism pathway involves the synthesis and degradation of pyrimidine nucleotides and plays an important role in DNA and RNA synthesis (Yang et al., 2024). The valine, leucine and isoleucine biosynthesis pathway is mainly involved in the biosynthesis of three amino acids, including valine, leucine and isoleucine, which are essential for a variety of cellular functions, including protein synthesis, energy metabolism and gene expression regulation (Hassan et al., 2023). Fructose and mannose metabolism is the metabolic process of fructose and mannose. The metabolism of the two is essential for maintaining the energy balance of cells (Lieu et al., 2021). In summary, genistein supplementation can lead to intestinal microorganisms enhancing Aminoacyl-tRNA biosynthesis, Pentose phosphate pathway, and Pyrimidine metabolism through the gut-liver axis, promoting the synthesis of protein and nucleic acid in the body, maintaining whole-body carbon–nitrogen metabolic homeostasis, and reducing Histidine metabolism, Phenylalanine, tyrosine and tryptophan biosynthesis, and Vitamin B6 metabolism, reducing the synthesis of substances such as histamine, tryptophan, and vitamin B6, thereby reducing body fat deposition and reducing inflammatory response. In addition, feeding with genistein can also lead to intestinal microorganisms enhancing Fructose and mannose metabolism, Amino sugar and nucleotide sugar metabolism, and Pentose phosphate pathway through the intestinal-muscle axis, promoting the metabolism of carbohydrates such as amino sugars and fructose to provide energy, maintain the body’s energy balance, and reduce Histidine metabolism, Phenylalanine, tyrosine and tryptophan biosynthesis, Valine, leucine and isoleucine biosynthesis, etc., reducing the metabolic process of histamine, tryptophan, and some branched-chain amino acids, thereby reducing the occurrence of inflammatory response in the body and ensuring host health.

## 5 Conclusion

The addition of genistein to the diet of finishing pigs had no significant effect on growth performance, but it could slightly increase the species relative abundance of intestinal flora, change the composition ratio of microbial communities, and did not affect the uniformity of species distribution. In addition, genistein supplementation had an effect on intestinal flora and metabolic pathways of liver and lumbar muscle, mainly manifested in a significant increase in the relative abundance of *Firmicutes* and a significant decrease in the relative abundance of *Bacteroidetes*. It also enhanced the aminoacyl-tRNA biosynthesis, Pentose phosphate pathway, Pyrimidine metabolism and other pathways through the gut-liver axis, promoting the synthesis of proteins and nucleic acids in the body and maintaining whole-body carbon–nitrogen metabolic homeostasis. At the same time, it enhanced the Fructose and mannose metabolism, Amino sugar and nucleotide sugar metabolism, Pentose phosphate pathway and other pathways through the gut-muscle axis, promoting the metabolism of sugar substances such as amino sugars and fructose to provide energy and maintain energy balance in the body. Correlation analysis showed that genistein supplementation may affect host phenotypic values by changing the relative abundance of bacterial genera related to phenotypes, but the specific regulatory mechanism needs further study.

We observed a higher *Firmicutes/Bacteroidetes* ratio and simultaneously activated aminoacyl-tRNA biosynthesis and pentose-phosphate pathways in the liver. In finishing pigs fed 160 mg/kg genistein, Hou et al. (2023) reported the same elevation of *Firmicutes* and a 30% increase in caecal butyrate, which was functionally linked to higher AMPK phosphorylation and lower hepatic gluconeogenesis (Hou et al., 2023). Butyrate is known to up-regulate G-protein-coupled receptor 41/43 in hepatocytes, thereby sparing glucose carbon for nucleotide and amino-acid biosynthesis—a metabolic signature that matches our targeted metabolome. Genistein reduced the relative abundance of LPS-producing taxa (e.g., *Escherichia-Shigella*) in our study. Guevara-Cruz et al. (2020) demonstrated that a 0.2% genistein intervention in obese humans lowered serum LPS by 35% and improved insulin sensitivity through decreased TLR4-NF-KB signaling (Guevara-Cruz et al., 2020). A similar anti-endotoxaemia mechanism likely operates in pigs and accounts for the tight negative correlation we observed between LPS-associated genera and host phenotypic indices.

## Data Availability

The data presented in this study are publicly available. This data can be found here: https://www.ncbi.nlm.nih.gov/sra, accession PRJNA1347656.
